# Evaluation of Audiometric Test Results to Determine Hearing Impairment in Patients with Rheumatoid Arthritis: Analysis of Data from the Korean National Health and Nutrition Examination Survey

**DOI:** 10.1371/journal.pone.0164591

**Published:** 2016-10-13

**Authors:** Hyemin Jeong, Young-Soo Chang, Sun Young Baek, Seon Woo Kim, Yeong Hee Eun, In Young Kim, Jaejoon Lee, Eun-Mi Koh, Hoon-Suk Cha

**Affiliations:** 1 Division of Rheumatology, Department of Internal Medicine, Samsung Medical Center, Sungkyunkwan University School of Medicine, Seoul, South Korea; 2 Department of Otorhinolaryngology, The Armed Forces Daejeon Hospital, Daejeon, South Korea; 3 Biostatic and Clinical Epidemiology Center, Samsung Medical Center, Seoul, South Korea; University of Catanzaro, ITALY

## Abstract

This study aimed to evaluate the association between rheumatoid arthritis (RA) and hearing impairment in the Korean adult population. Audiometric and laboratory test data from the 2010–2012 Korean National Health and Nutrition Examination Survey (KNHANES) were used for analysis. The relationship between RA and hearing impairment was analyzed, adjusting for various known risk factors associated with hearing impairment. RA was defined in the questionnaire as “RA diagnosed by a physician (yes/no) through a standardized interview.” We defined hearing impairment according to 2 categories of frequency (low/mid and high) as follows (average values in kHz): low/mid frequency, 0.5, 1.0, and 2.0, and high frequency, 3.0, 4.0, and 6.0. Of the subjects, 15,158 (weighted n = 32,035,996) completed the audiometric tests. The overall weighted prevalence of RA was 1.5%. The prevalence of hearing impairment was higher in the subjects with RA than in those without RA, in both, the low/mid- and high-frequency categories (21.1% vs 7.5%, p < 0.001 and 43.3% vs. 26.2%, p < 0.001, respectively). In the multivariable logistic analysis, RA (odds ratios [OR] 1.47, 95% confidence interval [CI] 1.05–2.06, p = 0.025) was an independent risk factor of low/mid-frequency hearing impairment along with age (OR 1.12, 95% CI 1.12–1.13, p < 0.001), current smoking (OR 1.27, 95% CI 1.03–1.56, p = 0.026), and college graduation (OR 0.53, 95% CI 0.39–0.72, p < 0.001). In the multivariable analysis of high-frequency hearing impairment, RA did not show any association with hearing impairment. This study suggests that RA is associated with low/mid-frequency hearing impairment after adjustment for various known risk factors. Further study is needed to verify the hearing impairment in RA.

## Introduction

Rheumatoid arthritis (RA) is a systemic chronic inflammatory disease that affects approximately 1% of the worldwide population [1,2]. RA affects synovitis extensively, resulting in erosions of the articular cartilage and marginal bone with joint destruction. Moreover, it is associated with progressive disability, systemic complications, early death and socioeconomic costs [3]. The systemic consequences of RA include heart, lung, liver, brain, skin, and eye involvement [4]. Hearing impairment is a common problem in the general population, and the effects of hearing loss are profound, with consequences in the social, functional, and psychological well-being of the persons affected. Severity of hearing impairment is significantly associated with having a hearing-related handicap and with self-reported communication difficulties [5]. The association between hearing impairment and autoimmune disease was first described by McCabe in 1979 [6]. After this report, other studies have demonstrated that the inner ear is a susceptible target of an autoimmune response and that sensorineural hearing loss can occur in complications of various autoimmune diseases including RA, ankylosing spondylitis, systemic lupus erythematous, Behcet’s disease, and psoriatic arthritis [7–12].

The association between RA and hearing impairment has been reported. Raut et al. performed a prospective case–control study to determine the association between RA and hearing impairment and the result showed a significant hearing impairment at 500 Hz, 1.0 kHz, and 2.0 kHz in patients with RA [7]. Ozcan et al. documented in a case-control study that the prevalence of hearing impairment was significantly higher in patients with RA [8]. Although several studies that addressed hearing impairment in RA subjects have been published, they were performed with consideration of only age and sex. In addition, a previous study reported that subjects with RA did not show any objective hearing threshold difference from subjects without RA [13]. Therefore, the association between RA and hearing impairment is still controversial, and it has not been fully determined in an analysis performed with adjustment for various possible factors. Age, sex, obesity, noise exposure, smoking, alcohol, education level, cardiovascular risk factors, and dietary supplements such as vitamin D have been reported as factors associated with hearing impairment [14–19].

The aim of this study was to evaluate the association between RA and hearing impairment by adjusting for various possible factors associated with hearing impairment in the Korean adult population by using data from the 2010–2012 Korea National Health and Nutrition Examination Survey (KNHANES).

## Methods

### Study population and data collection

The KNHANES is a nationwide survey that has been conducted periodically by the Korea Centers for Disease Control and Prevention to investigate the health and nutritional statuses of the Korean population. It assesses the general health and nutrition statuses of populations in South Korea through interviews about health and nutrition, and basic health assessments. Participants were selected by using the proportional allocation-systematic sampling method with multistage stratification to derive a representative Korean population. Although individual participants were not equally representative of the Korean population, this survey provides representative estimates of the noninstitutionalized Korean civilian population by using the power of sample weight. Every year, 10,000 to 12,000 individuals in about 3,800 households are selected from a panel based on the National Census Data. The participation rates of the selected households in the past several cycles of the KNHANES have been high, ranging from 79% to 84%. Written informed consent was obtained from all the participants before completing the survey.

To collect information on demographic variables, a standardized interview was performed by a professional investigator by using an established questionnaire, in the homes of the participants. The established questionnaire consisted of the demographic and socioeconomic characteristics of the subjects. Data on age, sex, smoking status, alcohol drinking, educational level, and occupational noise exposure were collected. *Heavy alcohol use* was defined as consuming alcohol more than four times per week during the month before the interview. *Occupational exposure* was defined as a history of longer than 3 months of exposure to loud noise at work that required speaking in a loud voice to be heard. *RA* was defined in the questionnaire as “RA diagnosed by a physician (yes/no) through a standardized interview.” The question was, “Was your RA diagnosed by RA a physician?” The interview was conducted individually by a trained professional investigator. Information on hypertension (yes/no) and diabetes mellitus (yes/no) that were diagnosed by a physician were also collected. Height and weight were assessed by using standardized techniques and equipment. Height was measured to the nearest 0.1 cm by using a portable stadiometer (Seriter, Bismarck, ND, USA). Weight was measured to the nearest 0.1 kg by using a Giant-150N calibrated balance-beam scale (Hana, Seoul, Korea). Body mass index (BMI) was calculated by dividing weight by the square of height (kg/m^2^). Systolic and diastolic blood pressures were measured while the patient was seated, by using the standard methods, that is, with a sphygmomanometer. Three measurements were recorded for all the subjects at 5-min intervals, and the average of the second and third measurements were used in the analysis. This study was approved by the interstitial review board of Samsung Medical Center.

### Laboratory methods

Blood samples were collected in the morning after fasting for at least 8 hours. In laboratory tests, total cholesterol (mg/dl), triglyceride (TG), serum creatinine, and serum vitamin D levels were measured by using the Hitachi Automatic Analyzer 7600 (Hitachi, Tokyo, Japan). Estimated glomerular filtration rate (eGFR) was calculated by using the abbreviated equation from the Modification of Diet in Renal Disease study as follows: eGFR (mL/min/1.73 m^2^) = 175 × (S_cr_/88.4, μmol/l)^−1.154^ × Age^−0.203^ × 0.742 (if female) [20].

### Audiometric measurement

The pure-tone air-conduction threshold was obtained in a soundproof booth by using an automatic audiometer (GSI SA-203, Entomed Diagnostics AB, Lena Nodin, Sweden). Trained otolaryngologists collected data independently for each ear at the following 6 frequencies: 0.5, 1.0, 2.0, 3.0, 4.0, and 6.0 kHz. All audiometric tests were performed under the supervision of an otolaryngologist. We determined hearing impairment according to 2 categories of frequency (low/mid, high). *Low/mid-frequency pure-tone threshold* was defined as the average of air-conduction hearing thresholds measured at 0.5, 1.0, and 2.0 kHz. *High-frequency pure-tone threshold* was defined as the average of air-conduction hearing thresholds measured at 3.0, 4.0, and 6.0 kHz. Based on data from large-population studies that showed that hearing abilities for frequencies of ≥3 kHz are the earliest and most severely affected, [21] we assessed the prevalence of both low/mid- and high-frequency hearing impairments. *Hearing impairment* was defined as an unaided pure-tone threshold level of ≥25 decibels (dB) for the superior ear. A 4-mm 0°-angled rigid endoscope attached to a charge-coupled device camera was used to perform endoscopic examinations in the study participants. Subjects with tympanic membrane perforation; cholesteatoma, including a retraction pocket; and otitis media with effusion were excluded from the study.

### Statistical analyses

To reflect representative estimates of the noninstitutionalized Korean civilian population, the survey sample weights, which were calculated by taking into account the sampling rate, response rate, and age/sex proportions of the reference population (2005 Korean National Census registry), were applied in all of the analyses. Potential associated factors, including age, sex, current smoking, heavy alcohol use, educational level, occupational noise exposure, BMI, hypertension, diabetes, total cholesterol level, serum vitamin D level, eGFR of <60 mL/min/1.73 m^2^, and RA, were evaluated by using a univariable analysis. Only variables with a p value of ≤0.2 were selected for the multivariable analysis with the logistic regression model. In the logistic regression analysis, we calculated the adjusted odds ratios (ORs) with the 95% confidence intervals (CIs) for hearing impairment. A subgroup analysis with stratification according to sex was also performed. By using the Bonferroni method, p values were corrected. Statistical analyses were performed by using SAS version 9.4 (SAS Institute, Cary, NC, USA). All p values were two-sided, and p values of <0.05 were considered statistically significant.

## Results

### Demographic and clinical characteristics of the study population

In total, 18,066 adults aged ≥19 years participated in the 2010–2012 KNHANES. Among the participants, those with ear pathologies in the endoscopic examination or missing data in the audiometric and laboratory tests were excluded. For this study, 15,158 subjects were analyzed. The weighted (weighted n = 32,035,996) demographic and clinical characteristics of the study population are presented in Table 1. The mean age of the participants was 44.6 years, and the percentage of females was 49.7%. The prevalence rates of current smoking and occupational noise exposure were 27.0% and 13.5%, respectively. Among 15,158 subjects, 297 (weighted n = 480,727) participants were RA and the weighted prevalence rate of RA was 1.5%. The prevalence rates of low/mid- and high-frequency hearing impairments in the Korean population in this study were 7.7% and 26.5%, respectively.

**Table 1:** Weighted baseline characteristics of the study population (unweighted, n = 15,158; weighted, n = 32,035,996).

Variables	
Age, years	44.6 ± 0.2
Female (%)	49.7
Current smoking (%)	27.0
Heavy alcohol use (%)	7.6
College graduation (%)	33.1
Occupational noise exposure (%)	13.5
Body mass index (kg/m^2^)	23.7 ± 0.0
Systolic blood pressure, mmHg	118.0 ± 0.2
Diastolic blood pressure, mmHg	76.5 ± 0.2
Hypertension (%)	16.2
Diabetes (%)	6.0
Total serum cholesterol, mg/dL	187.8 ± 0.4
Serum triglyceride, mg/dL	133.4 ± 1.2
Serum creatinine, mg/dL	0.84 ± 0.0
Serum vitamin D, ng/mL	17.3 ± 0.1
eGFR, ml/min/1.73m^2^	90.3 ± 0.2
Chronic kidney disease (eGFR < 60)	2.5
Rheumatoid arthritis (%)	1.5
Low-/mid-frequency hearing impairment (%)	7.7
High-frequency hearing impairment (%)	26.5

Continuous variables are expressed as mean ± standard error of the mean.

eGFR: estimated glomerular filtration rate; “Heavy alcohol use”: consuming alcohol more than four times per week during the month before the interview; “Occupational noise exposure”: a history of >3 months of loud noise at work that required speaking in a loud voice to be heard.

### Clinical characteristics according to the presence of RA

Clinical characteristics were analyzed according to the presence of RA (Table 2). The subjects with RA were older and more female predominant than the subjects without RA. The weighted prevalence of heavy alcohol use and college graduation was less common in the subjects with RA. On the contrary, subjects with hypertension, diabetes, and eGFRs of <60 ml/min/1.73 m^2^ were more prevalent in the RA group. The weighted prevalence rates of low/mid- (21.1% vs 7.5%, p < 0.001) and high-frequency hearing impairments (43.3% vs 26.2%, p < 0.001) were higher in the subjects with RA.

**Table 2:** Demographic and clinical characteristics according to the presence or absence of RA in the Korean adult population (2010–2012 KNHANES).

	RA (Weighted n = 480,727)	Without RA (Weighted n = 31,555,269)	p Value
Age, years	56.7 ± 1.1	44.5 ± 0.2	<0.001
Female (%)	75.4	49.3	<0.001
Current Smoking (%)	10.0	27.2	<0.001
Heavy alcohol use (%)	3.5	7.7	0.025
College graduation (%)	16.5	33.3	<0.001
Occupational noise exposure (%)	10.6	13.5	0.307
Body mass index (kg/m^2^)	23.8 ± 0.2	23.7 ± 0.0	0.768
Systolic blood pressure, mmHg	122.7 ± 1.3	117.9 ± 0.2	<0.001
Diastolic blood pressure, mmHg	76.5 ± 0.7	76.5 ± 0.2	0.902
Hypertension (%)	27.9	16.0	<0.001
Diabetes (%)	11.7	5.9	<0.001
Total serum cholesterol, mg/dL	188.2 ± 2.5	187.8 ± 0.4	0.861
Serum triglyceride, mg/dL	128.2 ± 6.5	133.4 ± 1.3	0.434
Serum creatinine, mg/dL	0.80 ± 0.02	0.84 ± 0.00	0.017
Serum vitamin D,ng/mL	18.3 ± 0.5	17.3 ± 0.1	0.037
eGFR <60ml/min/1.73m^2^ (%)	7.4	2.4	<0.001
Low-/mid-frequency hearing impairment (%)	21.1	7.5	<0.001
High-frequency hearing impairment (%)	43.3	26.2	<0.001

Continuous variables are expressed as mean ± standard error of the mean.

RA, rheumatoid arthritis; eGFR, estimated glomerular filtration rate

“Heavy alcohol use”: consuming alcohol more than four times per week during the month before the interview; “Occupational noise exposure”: a history of >3 months of loud noise at work that required speaking in a loud voice to be heard.

### Factors associated with low/mid-frequency hearing impairment

In the univariable logistic regression analysis for low/mid-frequency hearing impairment, age, sex, current smoking, heavy alcohol use, college graduation, hypertension, diabetes, total serum cholesterol level, serum vitamin D level, eGFR of <60 ml/min/1.73 m^2^, and RA were associated with low/mid-frequency hearing impairment (Table 3). In the multivariable analysis, RA was independently associated with low/mid-frequency hearing impairment, along with age, current smoking, and collage graduation. Age (OR 1.12, 95% CI 1.12–1.13, p < 0.001), current smoking (OR 1.27, 95% CI 1.03–1.56, p = 0.026), and RA (OR 1.47, 95% CI 1.05–2.06, p = 0.025) increased the likelihood of low/mid-frequency hearing impairment. College graduation (OR 0.53, 95% CI 0.39–0.72, p < 0.001) decreased the likelihood of low/mid-frequency hearing impairment.

**Table 3:** Logistic regression analysis to predict risk of low/mid-frequency hearing impairment in the Korean adult population.

			Univariable		Multivariable	
	Normal (Weighted n = 29,582,819)	Impaired (Weighted n = 2,453,177)	OR (95% CI)	p Value	OR (95% CI)	p Value
Age, years	42.8 ± 0.2	66.6 ± 0.5	1.13 (1.12–1.14)	<0.001	1.12 (1.12–1.13)	<0.001
Female (%)	49.3	54.4	1.23 (1.08–1.40)	0.002	0.96 (0.80–1.15)	0.667
Current smoking (%)	27.6	19.5	0.64 (0.54–0.75)	<0.001	1.27 (1.03–1.56)	0.026
Heavy alcohol use (%)	7.4	10.2	1.42 (1.15–1.75)	0.001	0.98 (0.75–1.28)	0.880
College graduation (%)	35.2	7.3	0.14 (0.11–0.19)	<0.001	0.53 (0.39–0.72)	<0.001
Occupational noise exposure (%)	13.4	14.8	1.13 (0.93–1.37)	0.223	-	-
Body mass index (kg/m^2^)	23.7 ± 0.1	23.9 ± 0.1	1.01 (0.99–1.03)	0.139	0.99 (0.97–1.02)	0.417
Hypertension (%)	13.7	45.6	5.27 (4.60–6.03)	<0.001	1.10 (0.94–1.29)	0.222
Diabetes (%)	5.0	17.3	3.96 (3.30–4.76)	<0.001	1.17 (0.94–1.46)	0.151
Total serum cholesterol, mg/dL	187.6 ± 0.4	190.7 ± 1.0	1.00 (1.00–1.01)	0.003	1.00 (0.99–1.00)	0.415
Serum vitamin D,ng/mL	17.2 ± 0.1	18.7 ± 0.3	1.04 (1.03–1.05)	<0.001	0.99 (0.98–1.01)	0.260
eGFR <60ml/min/1.73m2 (%)	1.8	11.0	6.85 (5.44–8.62)	<0.001	1.04 (0.80–1.35)	0.767
Rheumatoid arthritis (%)	1.3	4.1	3.33 (2.44–4.54)	<0.001	1.47 (1.05–2.06)	0.025

Continuous variables are expressed as mean ± standard error of the mean.

eGFR: estimated glomerular filtration rate; “Heavy alcohol use”: consuming alcohol more than four times per week during the month before the interview; “Occupational noise exposure”: a history of >3 months of loud noise at work that required speaking in a loud voice to be heard.

A subgroup analysis with stratification according to sex was performed. Among the male participants, age (OR 1.12, 95% CI 1.10–1.15, p < 0.001), college graduation (OR 0.57, 95% CI 0.38–0.84, p = 0.002), occupational noise exposure (OR 1.50, 95% CI 1.10–2.04, p = 0.007), and RA (OR 2.31, 95% CI 1.03–5.16, p = 0.039) were associated with low/mid-frequency hearing impairment in the multivariable analysis (S1 Table). In contrast to the male participants, the female participants did not show any significant association between RA and hearing impairment. Among the female subjects, age (OR 1.12, 95% CI 1.11–1.14, p < 0.001), college graduation (OR 0.51, 95% CI 0.27–0.95, p = 0.032), and hypertension (OR 1.34, 95% CI 1.05–1.71, p = 0.016) were associated with low/mid-frequency hearing impairment (S2 Table).

### Factors associated with high-frequency hearing impairment

In the univariable analysis for high-frequency hearing impairment, age, sex, heavy alcohol use, college graduation, occupational noise exposure, BMI, hypertension, diabetes, total serum cholesterol level, serum vitamin D level, eGFR of <60 ml/min/1.73 m^2^, and RA were associated with high-frequency hearing impairment (Table 4). In the multivariable analysis, age (OR 1.13, 95% CI 1.13–1.14, p < 0.001), and occupational noise exposure (OR 1.36, 95% CI 1.15–1.61, p < 0.001) increased the likelihood of high-frequency hearing impairment. Female sex (OR 0.27, 95% CI 0.24–0.31, p < 0.001) and college graduation (OR 0.59, 95% CI 0.50–0.70, p < 0.001) decreased the likelihood of high-frequency hearing impairment. RA was not associated with high-frequency hearing impairment in the multivariable analysis.

**Table 4:** Logistic regression analysis to predict risk of high-frequency hearing impairment in the Korean adult population.

			Univariable		Multivariable	
	Normal (Weighted n = 23,557,478)	Impaired (Weighted n = 8,478,518)	OR (95% CI)	p Value	OR (95% CI)	p Value
Age, years	39.1 ± 0.2	59.9 ± 0.3	1.13 (1.12–1.13)	<0.001	1.13 (1.13–1.14)	<0.001
Female (%)	53.6	38.8	0.55 (0.50–0.60)	<0.001	0.27 (0.24–0.31)	<0.001
Current smoking (%)	27.1	26.6	0.98 (0.88–1.09)	0.642	-	-
Heavy alcohol use (%)	5.7	13.0	2.48 (2.14–2.87)	<0.001	1.19 (0.98–1.45)	0.077
College graduation (%)	39.8	14.3	0.25 (0.22–0.29)	<0.001	0.59 (0.50–0.70)	<0.001
Occupational noise exposure (%)	12.6	17.4	1.53 (1.34–1.75)	<0.001	1.36 (1.15–1.61)	<0.001
Body mass index (kg/m^2^)	23.6 ± 0.1	24.0 ± 0.1	1.04 (1.02–1.05)	<0.001	0.99 (0.98–1.02)	0.759
Hypertension (%)	9.4	34.8	5.13 (4.61–5.71)	<0.001	1.02 (0.88–1.19)	0.811
Diabetes (%)	3.3	13.2	4.41 (3.76–5.17)	<0.001	1.04 (0.85–1.27)	0.695
Total serum cholesterol, mg/dL	186.5 ± 0.5	191.6 ± 0.7	1.00 (1.00–1.01)	<0.001	0.99 (0.99–1.00)	0.349
Serum vitamin D, ng/mL	16.7 ± 0.1	19.0 ± 0.2	1.06 (1.06–1.07)	<0.001	1.01 (0.99–1.02)	0.352
eGFR < 60 ml/min/1.73 m^2^ (%)	0.9	6.8	7.90 (6.17–10.13)	<0.001	1.14 (0.84–1.54)	0.404
Rheumatoid arthritis (%)	1.2	2.5	2.16 (1.65–2.81)	<0.001	0.99 (0.74–1.32)	0.926

Continuous variables are expressed as mean ± standard error of the mean.

eGFR: estimated glomerular filtration rate; “Heavy alcohol use”: consuming alcohol more than four times per week during the month before the interview; “Occupational noise exposure”: a history of >3 months of loud noise at work that required speaking in a loud voice to be heard.

A subgroup analysis with stratification according to sex was performed. Among the male participants, age (OR 1.13, 95% CI 1.12–1.14, p < 0.001), heavy alcohol use (OR 1.27, 95% CI 1.00–1.62, p = 0.050), college graduation (OR 0.66, 95% CI 0.53–0.82, p < 0.001), and occupational noise exposure (OR 1.54, 95% CI 1.21–1.97, p < 0.001) were associated with high-frequency hearing impairment in the multivariable analysis (S3 Table). Among the female participants, age (OR 1.14, 95% CI 1.13–1.16, p < 0.001) and college graduation (OR 0.39, 95% CI 0.27–0.57, p < 0.001) were associated with high-frequency hearing impairment (S4 Table). RA was not significantly associated with high-frequency hearing impairment in both the male and female participants.

## Discussion

By using data from a large-scale nationwide survey conducted by the Korean government, we found that the prevalence of hearing impairment was higher in patients with RA than in those without RA. In addition, RA was significantly associated with low/mid-frequency hearing impairment in the multivariable analysis after adjustment for various possible factors known as associated with hearing impairment. Our data were consistent with those of the previous research that showed that subjects with RA were more likely to have a low/mid-frequency hearing impairment [8,22,23]. Cochlear-type sensorineural hearing impairment, including RA, has been reported to specifically affect the lower-frequency hearing ability [7]. By contrast, other studies reported that RA is associated with high-frequency hearing impairment [24,25]. Considering that adjustment of comorbid conditions was not performed in this study, interpretation of our data has limitation. As RA is associated with increased incidence of various comorbidities, including hypertension, diabetes, and chronic kidney disease, these comorbidities are also associated with hearing impairment. In the present study, although high-frequency hearing impairment (43.3%) was more common than low/mid-frequency hearing impairment (21.1%), RA did not show a significant correlation with high-frequency hearing impairment in the multivariable analysis. Hearing loss has been reported to develop first at high frequencies, and various factors such as age, sex, smoking, or occupational noise exposure are related to high-frequency hearing loss [26–29]. This suggests that high-frequency hearing impairment is affected by various factors and in a quantitative manner from early in life and that is more related with damage to lower-frequency hearing ability. Low frequency, the pure tone average of hearing thresholds at 0.5, 1.0, and 2.0 kHz, is known as the range of the human voice and is widely accepted as a parameter for evaluating degree of hearing impairment [30]. Therefore, the association between the occurrence of RA and low/mid-frequency hearing impairment is worthy of notice, and we need to pay more attention to the possible presence of hearing impairment and hearing-related handicap in patients with RA.

The pathophysiological mechanism of sensorineural hearing loss in RA is not fully understood. It has been suggested that damage such as vasculitis or neuritis may affect the inner ear structures. Moreover, autoantibodies create an immune complex-mediated vasculitis against inner ear structures or may cause sensorineural hearing impairment in RA [22,31]. Ototoxicity of medication used for the treatment of RA, including non-steroidal anti-inflammatory drugs, antimalarial agents, and some other disease-modifying anti-rheumatic drugs can affect the auditory system [32–34]. In addition, conductive hearing loss related with joint destruction in ossicles has been suggested as a possible pathophysiological mechanism of hearing impairment in subjects with RA [8,35,36]. One explanation of conductive hearing loss in RA patients is the synovial involvement of the middle ear joints. As the incudomalleolar and incudostapedial joints are true diarthroses, these structures might be susceptible to the autoimmune arthritis in subjects with RA.

The incidence of cardiovascular disease is well known to increase among RA patients, as compared with the general population. Cardiovascular disease mortality is increased by 50% in RA patients compared with the general population [37]. Previous studies have found that the increased risk of cardiovascular disease in patients with RA is comparable with the risk in patients with type 2 diabetes mellitus [38,39]. As high disease activity is associated with increased mortality in patients with RA [40], the European League against Rheumatism recommends that adequate control of disease activity is necessary to lower cardiovascular risk [41]. The systemic inflammation in RA is associated with cardiovascular events. Atherosclerosis and RA share many common inflammatory pathways. High levels of tumor necrosis factor (TNF) and IL-6 associated with RA are also significantly associated with the development of atherosclerosis [42,43]. In addition, the altered structure and function of platelets interact with the synovial membrane and vascular wall, resulting in arthritis and atherosclerosis in patients with RA [44]. Hearing impairment is associated with cardiovascular risk factors such as smoking, hypertension, diabetes, and dyslipidemia [16,19]. Hearing impairment, especially for low frequency, was related to cardiovascular disease events [16]. High systolic blood pressure was related with hearing loss in the low and mid frequencies in elderly women [45]. Low level of high-density lipoprotein cholesterol is associated with hearing impairment at 2000 and 4000 Hz. Friedland et al. reported that lower-frequency hearing loss was a predictor of cardiovascular diseases such as stroke, transient ischemic attack, peripheral vascular disease and myocardial infarction [46]. In animal models, hypertension and dyslipidemia induced degeneration of stria vascularis and auditory threshold elevation [47,48]. Kakarlapudi et al. reported that sensorineural hearing loss was more common in patients with diabetes than in control nondiabetic subjects, and that severity of hearing loss correlated with diabetes progression as reflected in serum creatinine levels [49]. Hearing impairment, especially for low/mid frequencies, was related with diabetes severity [50]. Microangiopathy in the stria vascularis that is caused by diabetes has been considered as an important factor of the development of vestibular-cochlear disorders in the inner ear [51,52]. In addition, a recent study reported that the prevalence of undiagnosed diabetes increased in RA [53]. Although the patients with undiagnosed diabetes in the present study could not be ascertained, we included the diagnosis of diabetes in the analysis. Hearing impairment was more common in the patients with diabetes for longer durations [54]. Considering that patients with undiagnosed diabetes may be in the early stage of the disease and that serum creatinine level is included in the analysis, the effect of the possible presence of patients with undiagnosed diabetes in the RA group may be limited. Taken together, systemic inflammation in RA may cause systemic and local vascular pathologies, including those in the vertebrobasilar system, which would result in cardiovascular disease and hearing impairment.

In the present study, hearing impairment was associated with aging and lower educational level in the cases with low/mid- and high-frequency hearing impairments. Increasing age and occupational noise exposure are known contributing factors to hearing impairment across the frequency spectrum [29,55,56]. Individuals with higher educational levels might be more likely to work in better-quality environment with less noise exposure and have a better access to health care. Therefore, an inverse association between educational level and hearing impairment was observed in both the low/mid and high frequencies.

In the present study, sex-related differences were observed in the factors associated with hearing impairment. In the female participants, occupational noise exposure was not related to hearing impairment in the multivariable analysis. A previous study showed that sex gap was found in occupational hearing loss, in favor of female [57]. The female sex hormone estrogen may play a role in preserving hearing [58]. A previous study reported that differences in auditory brainstem response between men and women, with the women having shorter latencies and larger amplitudes than the men [59]. Hormonal changes accompanying menopause may account for the sex-related difference in hearing. The auditory brainstem response latencies in younger women are shorter than those in younger men, but this relationship changes with age and postmenopausal women present almost the same values as men of the same age [60]. Postmenopausal women have better hearing abilities if they undergo hormone replacement therapy [61] and Turner`s syndrome (45,X) showed early presbycusis [62]. In an animal study, severe progressive hearing loss occurred in ERß knockout mice [63]. In the multivariable analysis, RA was not significantly related to the hearing impairment in the females. It may be explained by the fact that age might be a more potent risk factor of hearing impairment in females than in males, considering the rapid decrease in estrogen level in older menopausal women.

The present study has some limitations. First, the diagnosis of RA was dependent on the information provided by the participants in the interview. In this study, specific criteria were not applied for the definition of RA. This is one major limitation of nationwide surveys of this type. The prevalence of RA in this study was 1.5%. Considering that the prevalence of RA is approximately 1% of the worldwide population [1,2], the prevalence of RA in the present study might have been overestimated. Second, because our study used data from a nationwide survey, we could not access the disease activity or medications for RA and were unable to evaluate the association between the disease activity of RA and hearing impairment. We cannot draw an inference as to causality due to the cross-sectional design of the study and the limitations about definition of RA. The strength of our study is that we analyzed large, nationally representative data of the Korean adult population. Audiometric test was performed to all participants. To the best of our knowledge, this is the first study to suggest the association between RA and low/mid-frequency hearing impairment after adjustment for previously reported risk factors of hearing impairment by using nationally representative data.

In conclusion, this study demonstrates the association between RA and hearing impairment in the Korean adult population. We found that RA specifically affects lower-frequency hearing abilities after adjusting for various possible factors known to be associated with hearing impairment. These findings suggest that individuals with RA might be at risk of hearing impairment and clinicians need to pay attention to the possibility of hearing impairment and screening for hearing loss in individuals with RA. In spite of limitations of our study, the data reported in this manuscript provide a comprehensive cross-sectional data of RA and audiometric test. Our results provide an initial analysis from a nation-wide survey. Further study is warranted to verify the hearing impairment in patients with RA based on specific classification criteria of RA. And studies about association between hearing impairment and disease severity of RA or medications for RA are also needed. We believe that present study will serve as a starting point for future researchers to investigate the association of hearing impairment and RA.

## Supporting Information

S1 Table Logistic regression analysis to predict risk of of low/mid-frequency hearing impairment in the Korean male adult population. Continuous variables are expressed as mean ± standard error of the mean. eGFR: estimated glomerular filtration rate; “Heavy alcohol use”: consuming alcohol more than four times per week during the month before the interview; “Occupational noise exposure”: a history of >3 months of loud noise at work that required speaking in a loud voice to be heard.
(DOCX)

S2 Table Logistic regression analysis to predict risk of low/mid-frequency hearing impairment in the Korean female adult population. Continuous variables are expressed as mean ± standard error of the mean. eGFR: estimated glomerular filtration rate; “Heavy alcohol use”: consuming alcohol more than four times per week during the month before the interview; “Occupational noise exposure”: a history of >3 months of loud noise at work that required speaking in a loud voice to be heard.
(DOCX)

S3 Table Logistic regression analysis to predict risk of high-frequency hearing impairment in the Korean male adult population. Continuous variables are expressed as mean ± standard error of the mean. eGFR: estimated glomerular filtration rate; “Heavy alcohol use”: consuming alcohol more than four times per week during the month before the interview; “Occupational noise exposure”: a history of >3 months of loud noise at work that required speaking in a loud voice to be heard.
(DOCX)

S4 Table Logistic regression analysis to predict risk of high-frequency hearing impairment in the Korean female adult population. Continuous variables are expressed as mean ± standard error of the mean. eGFR: estimated glomerular filtration rate; “Heavy alcohol use”: consuming alcohol more than four times per week during the month before the interview; “Occupational noise exposure”: a history of >3 months of loud noise at work that required speaking in a loud voice to be heard.
(DOCX)
