# Supplementary material for: Evaluation of Audiometric Test Results to Determine Hearing Impairment in Patients with Rheumatoid Arthritis: Analysis of Data from the Korean National Health and Nutrition Examination Survey
Source: PLoS One. 2016 Oct 13;11(10):e0164591. doi: 10.1371/journal.pone.0164591 (PMC5063362; doi:10.1371/journal.pone.0164591)
Supplement: S1 Table — Continuous variables are expressed as mean ± standard error of the mean. eGFR: estimated glomerular filtration rate; “Heavy alcohol use”: consuming alcohol more than four times per week during the month before the interview; “Occupational noise exposure”: a history of >3 months of loud noise at work that required speaking in a loud voice to be heard. (DOCX) [file pone.0164591.s001.docx]

**S1 Table. Logistic regression analysis to predict risk of of low/mid-frequency hearing impairment in the Korean male adult population**

|  | | | **Univariable** | | **Multivariable** | |
| --- | --- | --- | --- | --- | --- | --- |
|  | **Normal (Weighted n = 14,994,126)** | **Impaired (Weighted n = 1,117,790)** | **OR (95% CI)** | **p Value** | **OR (95% CI)** | **p Value** |
| Age, years | 42.3 ± 0.3 | 65.0 ± 0.8 | 1.13 (1.11–1.14) | <0.001 | 1.12 (1.10–1.15) | <0.001 |
| Current smoking (%) | 47.4 | 35.1 | 0.60 (0.48–0.75) | <0.001 | 1.12 (0.85–1.49) | 0.740 |
| Heavy alcohol use (%) | 12.5 | 19.8 | 1.73 (1.31–2.29) | <0.001 | 1.00 (0.72–1.41) | 1.969 |
| College graduation (%) | 38.4 | 12.5 | 0.23 (0.16–0.32) | <0.001 | 0.57 (0.38–0.84) | 0.002 |
| Occupational noise exposure (%) | 19.1 | 22.9 | 1.26 (0.96–1.65) | 0.115 | 1.50 (1.10–2.04) | 0.007 |
| Body mass index (kg/m^2^) | 24.2 ± 0.1 | 23.5 ± 0.1 | 0.93 (0.90–0.96) | <0.001 | 0.99 (0.94–1.04) | 1.047 |
| Hypertension (%) | 14.0 | 36.7 | 3.58 (2.85–4.48) | <0.001 | 0.87 (0.67–1.14) | 0.514 |
| Diabetes (%) | 5.7 | 17.1 | 3.44 (2.57–4.60) | <0.001 | 1.21 (0.84–1.73) | 0.475 |
| Total serum cholesterol, mg/dL | 187.7 ± 0.6 | 183.3 ± 1.5 | 0.99 (0.99–0.99) | 0.011 | 0.99 (0.99–1.00) | 0.980 |
| Serum vitamin D, ng/mL | 18.1 ± 0.2 | 19.6 ± 0.3 | 1.04 (1.02–1.06) | <0.001 | 0.99 (0.98–1.02) | 1.361 |
| eGFR < 60 ml/min/1.73 m^2^ (%) | 1.9 | 10.7 | 6.21 (4.20–9.18) | <0.001 | 1.11 (0.75–1.65) | 1.105 |
| Rheumatoid arthritis (%) | 0.6 | 2.3 | 3.73 (1.69–8.23) | <0.001 | 2.31 (1.03–5.16) | 0.039 |

Continuous variables are expressed as mean ± standard error of the mean.

eGFR: estimated glomerular filtration rate; “Heavy alcohol use”: consuming alcohol more than four times per week during the month before the interview; ‟Occupational noise exposure”: a history of >3 months of loud noise at work that required speaking in a loud voice to be heard.
