# Supplementary material for: Evaluation of Audiometric Test Results to Determine Hearing Impairment in Patients with Rheumatoid Arthritis: Analysis of Data from the Korean National Health and Nutrition Examination Survey
Source: PLoS One. 2016 Oct 13;11(10):e0164591. doi: 10.1371/journal.pone.0164591 (PMC5063362; doi:10.1371/journal.pone.0164591)
Supplement: S2 Table — Continuous variables are expressed as mean ± standard error of the mean. eGFR: estimated glomerular filtration rate; “Heavy alcohol use”: consuming alcohol more than four times per week during the month before the interview; “Occupational noise exposure”: a history of >3 months of loud noise at work that required speaking in a loud voice to be heard. (DOCX) [file pone.0164591.s002.docx]

**S2 Table. Logistic regression analysis to predict risk of low/mid-frequency hearing impairment in the Korean female adult population**

|  | | | **Univariable** | | **Multivariable** | |
| --- | --- | --- | --- | --- | --- | --- |
|  | **Normal (Weighted n = 14,588,693)** | **Impaired (Weighted**  **n = 1,335,387)** | **OR (95% CI)** | **p Value** | **OR (95% CI)** | **p Value** |
| Age, years | 43.4 ± 0.2 | 67.9 ± 0.5 | 1.14 (1.12–1.15) | <0.001 | 1.12 (1.11–1.14) | <0.001 |
| Current smoking (%) | 7.3 | 6.4 | 0.87 (0.56–1.37) | 0.993 | - | - |
| Heavy alcohol use (%) | 2.2 | 2.2 | 0.98 (0.53–1.79) | 1.000 | - | - |
| College graduation (%) | 31.9 | 2.9 | 0.06 (0.04–0.11) | <0.001 | 0.51 (0.27–0.95) | 0.032 |
| Occupational noise exposure (%) | 7.5 | 8.0 | 1.09 (0.72–1.63) | 1.000 | - | - |
| Body mass index (kg/m^2^) | 23.2 ± 0.1 | 24.2 ± 0.1 | 1.07 (1.05–1.09) | <0.001 | 0.99 (0.96–1.02) | 0.759 |
| Hypertension (%) | 13.5 | 53.0 | 7.24 (5.88–8.91) | <0.001 | 1.34 (1.05–1.71) | 0.016 |
| Diabetes (%) | 4.3 | 17.4 | 4.65 (3.49–6.19) | <0.001 | 1.17 (0.84–1.62) | 0.590 |
| Total serum cholesterol, mg/dL | 187.4 ± 0.6 | 196.9 ± 1.4 | 1.01 (1.00–1.01) | <0.001 | 1.00 (0.99–1.00) | 1.000 |
| Serum vitamin D, ng/mL | 16.3 ± 0.1 | 18.0 ± 0.3 | 1.05 (1.03–1.06) | <0.001 | 0.99 (0.97–1.01) | 0.380 |
| eGFR < 60 ml/min/1.73 m^2^ (%) | 1.7 | 11.3 | 7.56 (5.24–10.90) | <0.001 | 1.01 (0.66–1.56) | 1.000 |
| Rheumatoid arthritis (%) | 2.0 | 5.7 | 3.02 (2.01–4.54) | <0.001 | 1.25 (0.81–1.92) | 0.515 |

Continuous variables are expressed as mean ± standard error of the mean.

eGFR: estimated glomerular filtration rate; “Heavy alcohol use”: consuming alcohol more than four times per week during the month before the interview; ‟Occupational noise exposure”: a history of >3 months of loud noise at work that required speaking in a loud voice to be heard.
