# Supplementary material for: Evaluation of Audiometric Test Results to Determine Hearing Impairment in Patients with Rheumatoid Arthritis: Analysis of Data from the Korean National Health and Nutrition Examination Survey
Source: PLoS One. 2016 Oct 13;11(10):e0164591. doi: 10.1371/journal.pone.0164591 (PMC5063362; doi:10.1371/journal.pone.0164591)
Supplement: S3 Table — Continuous variables are expressed as mean ± standard error of the mean. eGFR: estimated glomerular filtration rate; “Heavy alcohol use”: consuming alcohol more than four times per week during the month before the interview; “Occupational noise exposure”: a history of >3 months of loud noise at work that required speaking in a loud voice to be heard. (DOCX) [file pone.0164591.s003.docx]

**S3 Table. Logistic regression analysis to predict risk of high-frequency hearing impairment in the Korean male adult population**

|  | | | **Univariable** | | **Multivariable** | |
| --- | --- | --- | --- | --- | --- | --- |
|  | **Normal (Weighted n = 10,922,779)** | **Impaired (Weighted**  **n = 5,189,137)** | **OR (95% CI)** | **p Value** | **OR (95% CI)** | **p Value** |
| Age, years | 37.5 ± 0.3 | 57.2 ± 0.3 | 1.13 (1.12–1.14) | <0.001 | 1.13 (1.12–1.14) | <0.001 |
| Current smoking (%) | 49.4 | 40.3 | 0.69 (0.60–0.80) | <0.001 | 1.09 (0.88–1.34) | 0.748 |
| Heavy alcohol use (%) | 9.7 | 20.1 | 2.34 (1.94–2.82) | <0.001 | 1.27 (1.00–1.62) | 0.050 |
| College graduation (%) | 44.0 | 21.1 | 0.34 (0.29–0.40) | <0.001 | 0.66 (0.53–0.82) | <0.001 |
| Occupational noise exposure (%) | 17.5 | 23.3 | 1.43 (1.18–1.72) | <0.001 | 1.54 (1.21–1.97) | <0.001 |
| Body mass index (kg/m^2^) | 24.3 ± 0.1 | 23.9 ± 0.1 | 0.97 (0.95–0.99) | <0.001 | 0.99 (0.96–1.02) | 1.000 |
| Hypertension (%) | 8.9 | 29.3 | 4.21 (3.56–4.98) | <0.001 | 0.96 (0.75–1.22) | 1.000 |
| Diabetes (%) | 3.6 | 12.6 | 3.89 (3.00–5.05) | <0.001 | 1.00 (0.72–1.39) | 1.000 |
| Total serum cholesterol, mg/dL | 187.3 ± 0.8 | 187.7 ± 0.9 | 1.00 (0.99–1.00) | 1.000 | - | - |
| Serum vitamin D, ng/mL | 17.6 ± 0.2 | 19.5 ± 0.2 | 1.05 (1.04–1.07) | <0.001 | 1.00 (0.99–1.02) | 1.000 |
| eGFR < 60 ml/min/1.73 m^2^ (%) | 0.9 | 6.0 | 7.34 (4.76–11.31) | <0.001 | 1.28 (0.78–2.09) | 0.536 |
| Rheumatoid arthritis (%) | 0.55 | 1.11 | 2.02 (0.95–4.28) | 0.072 | 0.87 (0.41–1.87) | 1.000 |

Continuous variables are expressed as mean ± standard error of the mean.

eGFR: estimated glomerular filtration rate; “Heavy alcohol use”: consuming alcohol more than four times per week during the month before the interview; ‟Occupational noise exposure”: a history of >3 months of loud noise at work that required speaking in a loud voice to be heard.
