# Supplementary material for: Evaluation of Audiometric Test Results to Determine Hearing Impairment in Patients with Rheumatoid Arthritis: Analysis of Data from the Korean National Health and Nutrition Examination Survey
Source: PLoS One. 2016 Oct 13;11(10):e0164591. doi: 10.1371/journal.pone.0164591 (PMC5063362; doi:10.1371/journal.pone.0164591)
Supplement: S4 Table — Continuous variables are expressed as mean ± standard error of the mean. eGFR: estimated glomerular filtration rate; “Heavy alcohol use”: consuming alcohol more than four times per week during the month before the interview; “Occupational noise exposure”: a history of >3 months of loud noise at work that required speaking in a loud voice to be heard. (DOCX) [file pone.0164591.s004.docx]

**S4 Table. Logistic regression analysis to predict risk of high-frequency hearing impairment in the Korean female adult population**

|  | | | **Univariable** | | **Multivariable** | |
| --- | --- | --- | --- | --- | --- | --- |
|  | **Normal (Weighted n = 12,634,698)** | **Impaired (Weighted n = 3,289,382)** | **OR (95% CI)** | **p Value** | **OR (95% CI)** | **p Value** |
| Age, years | 40.5 ± 0.2 | 64.2 ± 0.4 | 1.15 (1.14–1.16) | <0.001 | 1.14 (1.13–1.16) | <0.001 |
| Current smoking (%) | 7.8 | 5.0 | 0.62 (0.45–0.86) | 0.002 | 1.23 (0.77–1.95) | 0.656 |
| Heavy alcohol use (%) | 2.3 | 1.9 | 0.85 (0.53–1.35) | 0.844 | - | - |
| College graduation (%) | 36.2 | 3.7 | 0.07 (0.05–0.09) | <0.001 | 0.39 (0.27–0.57) | <0.001 |
| Occupational noise exposure (%) | 7.4 | 8.0 | 1.10 (0.84–1.45) | 0.845 | - | - |
| Body mass index (kg/m^2^) | 23.0 ± 0.1 | 24.2 ± 0.1 | 1.09 (1.07–1.11) | <0.001 | 0.99 (0.96–1.03) | 1.000 |
| Hypertension (%) | 9.8 | 43.5 | 7.06 (5.95–8.38) | <0.001 | 1.06 (0.85–1.32) | 1.000 |
| Diabetes (%) | 3.2 | 14.3 | 5.12 (3.98–6.58) | <0.001 | 1.00 (0.72–1.39) | 1.000 |
| Total serum cholesterol, mg/dL | 185.8 ± 0.6 | 197.6 ± 0.9 | 1.01 (1.00–1.01) | <0.001 | 1.00 (0.99–1.00) | 0.446 |
| Serum vitamin D, ng/mL | 15.9 ± 0.1 | 18.2 ± 0.2 | 1.07 (1.05–1.08) | <0.001 | 1.01 (0.99–1.02) | 0.416 |
| eGFR < 60 ml/min/1.73 m^2^ (%) | 1.0 | 8.2 | 9.10 (6.31–13.13) | <0.001 | 1.00 (0.63–1.59) | 1.000 |
| Rheumatoid arthritis (%) | 1.7 | 4.6 | 2.81 (2.01–3.93) | <0.001 | 0.98 (0.68–1.42) | 1.000 |

Continuous variables are expressed as mean ± standard error of the mean.

eGFR: estimated glomerular filtration rate; “Heavy alcohol use”: consuming alcohol more than four times per week during the month before the interview; ‟Occupational noise exposure”: a history of >3 months of loud noise at work that required speaking in a loud voice to be heard.
